# The China birth cohort study (CBCS)

**DOI:** 10.1007/s10654-021-00831-8

**Published:** 2022-02-11

**Authors:** Wentao Yue, Enjie Zhang, Ruixia Liu, Yue Zhang, Chengrong Wang, Shen Gao, Shaofei Su, Xiao Gao, Qingqing Wu, Xiaokui Yang, Aris T. Papageorghiou, Chenghong Yin

**Affiliations:** 1 Department of Central Laboratory, Beijing Obstetrics and Gynecology Hospital, Capital Medical University, Beijing, People’s Republic of China; 2 Department of Ultrasound, Beijing Obstetrics and Gynecology Hospital, Capital Medical University, Beijing, People’s Republic of China; 3 Department of Reproductive Medicine, Beijing Obstetrics and Gynecology Hospital, Capital Medical University, Beijing, People’s Republic of China; 4 Nuffield Department of Women’s and Reproductive Health, University of Oxford, John Radcliffe Hospital, Oxford, UK

**Keywords:** Cohort study, Birth defects, Pregnancy, China

## Abstract

The China birth cohort study (CBCS) is a prospective longitudinal, mega-cohort study and the first national-based birth cohort study, aiming to establish a birth cohort covering representative geographical areas of the whole of China to investigate risk factors for birth defects and develop strategies for their reduction. Pregnant women who are of Chinese nationality, are 6–13^+6^ weeks of gestation, plan to attend the routine antenatal examination and deliver in the study site, and give their informed, written consent are eligible to participate in this study. All participants are followed-up through an in-person interview at 20–23^+6^ weeks and again at 28–33^+6^ weeks of gestation, and at delivery, respectively. CBCS has been divided into three phases from 20th November 2017 to 31st December 2021, and the first two phases have now been completed on 29th February 2020, enrolling 120 377 eligible pregnant women during this period. During the same period a total of 40 837 participants had been followed up to the end of pregnancy. Study recruitment will continue until December 2021 to achieve the target of 500 000 participants. Meanwhile, biological samples including peripheral blood, amniocytes, cord blood, placenta, or umbilical cord tissue have been collected from participants according to various conditions. The incidence of birth defects in this group is 2.5% and congenital heart disease is the most common type of birth defect seen so far. A website is in the advanced stages of planning, to allow seamless data transfer and facilitate collaboration with groups around the world.

**Supplementary Information:**

The online version contains supplementary material available at 10.1007/s10654-021-00831-8.

## Introduction

Birth defects are an important cause of infant mortality and lifelong disability, which result in tremendous burden to families and the society. According to the 2010 Global Burden of Disease (GBD) study, 6.4% of neonatal infant deaths are attributed to birth defects, which ranked 5th among all causes of death [1]. This burden appears not to be evenly distributed, rather it has been reported that the prevalence of all birth defects in live births ranges from a high of 8.2% to a low of 4.0% worldwide. In addition to lethal birth defects, it has been estimated that at least 3.2 million survivors may suffer from significant disability for life [2]. It is important to note that the impact of birth defects is particularly severe in middle- and low-income countries including China. According to the report of Chinese Birth Defect prevention in 2012, there are about 900,000 annual births that are affected by birth defects, accounting for 5.6% of all births [3]. At the same time, given the rapid economic development, industrialization and urbanization in China and beyond, concerns have been raised in relation to environmental pollution as a modifiable influence on pregnant women and, by extension, on the prevalence of birth defects [4]. Hence, a better understanding of demographic distributions and risk factors associated with birth defects is urgently needed to provide evidence for etiology and prevention. We believe there is an important need to determine these exposotypes at the population level as they may help us to understand how exposures affect the occurrence of birth defects at the individual and systems level, and can therefore lead to determining cause, effect, and susceptibilities. However, most of the previous evidence comes from high income settings in the USA and Europe, with studies rather insufficient and limited in Chinese populations [5–8].

The China birth cohort study (CBCS) is a prospective, longitudinal, mega-cohort study ultimately aiming at prevention of birth defects in China. The CBCS aims to establish a birth cohort of 500 000 pregnant women covering all regions of China from 20th November 2017 to 31st December 2021. Compared with other Chinese cohorts mentioned above, CBCS is the both the most recent and the first nation-wide birth cohort study in China and on completion will be one of the world’s largest birth cohort studies.

The main hypothesis of our research is that birth defects depend on many risk factors and exposures suffered even before pregnancy. Therefore, the objective is to establish and estimate the relationship between demographic, genetic, behavioural and environmental maternal exposures before and during pregnancy with birth defects. There are three major research interests: (1) Describing epidemiological characteristics of birth defects and other adverse outcomes; (2) Investigating the impacts of maternal exposure, including environmental determinants, on birth defects and other adverse outcomes (the exposotype); (3) Identifying risk factors specifically for congenital heart diseases, which constitute the most commonly diagnosed birth defect.

The design phase of the CBCS started in 2015 at the Beijing Obstetrics and Gynecology Hospital, Capital Medical University. A multidisciplinary scientific panel, including public health experts, obstetricians, ultrasound specialists, prenatal screening specialists, geneticists, environmentalists, and microbiologists, were invited over a number of cycles to design the study, including the research protocol, standard operating procedures, questionnaire design, data collection forms and methodology for follow-up, as well as to assess overall feasibility. In addition, the Chinese Ministry of Science and Technology has prioritised the establishment of the CBCS to further investigate these issues, and has provided non-financial as well as financial support (¥60 million / USD 8.3 million).

### Study cohort

Pregnant women are eligible to participate in the study if (1) they are of Chinese nationality; (2) they are 6–13^+6^ weeks of gestation at the time of recruitment, including both natural pregnancy and conception using assisted reproductive technologies; (3) they plan to attend routine antenatal examination and deliver in the study site, and plan to continue to live locally for more than one year; (4) they have no notifiable infectious diseases such as hepatitis B, syphilis and HIV; (5) they are able to understand the study and willing to give informed, written consent. It is possible for included women to withdraw from the study at any stage.

The large sample size of CBCS will provide adequate power to investigate not just associations but also potential causal effects of different exposures on birth defects. The sheer size of the study means that detailed planning is necessary to ensure feasibility, effectiveness of recruitment and follow-up, and standardization of data collection as well as biological sampling / processing; for this reason, CBCS has been divided into three phases.

- The first phase was a 5-month pilot study used to evaluate all elements, including data collection systems and implementation processes of the study, and assess variables collected in six research sites from 20th November 2017 to 31st March 2018.
- The second phase aims to evaluate the effectiveness of recruitment and follow-up procedures and the standardization of quality control procedures from 1st April 2018 to 29th February 2020.
- After the operationalization of these first two phases, and prior to the third phase, which aims to invite more research sites to enrol participants, we undertook this pre-planned interim assessment of all processes for the study. This is the subject of this report, and we were particularly interested to examine follow-up rates, and establish whether recruitment of more sites during the final phase (from 1st March 2020 to 31st December 2021) is required, thus ensuring that the study can be completed on time.

**Fig. 1:**
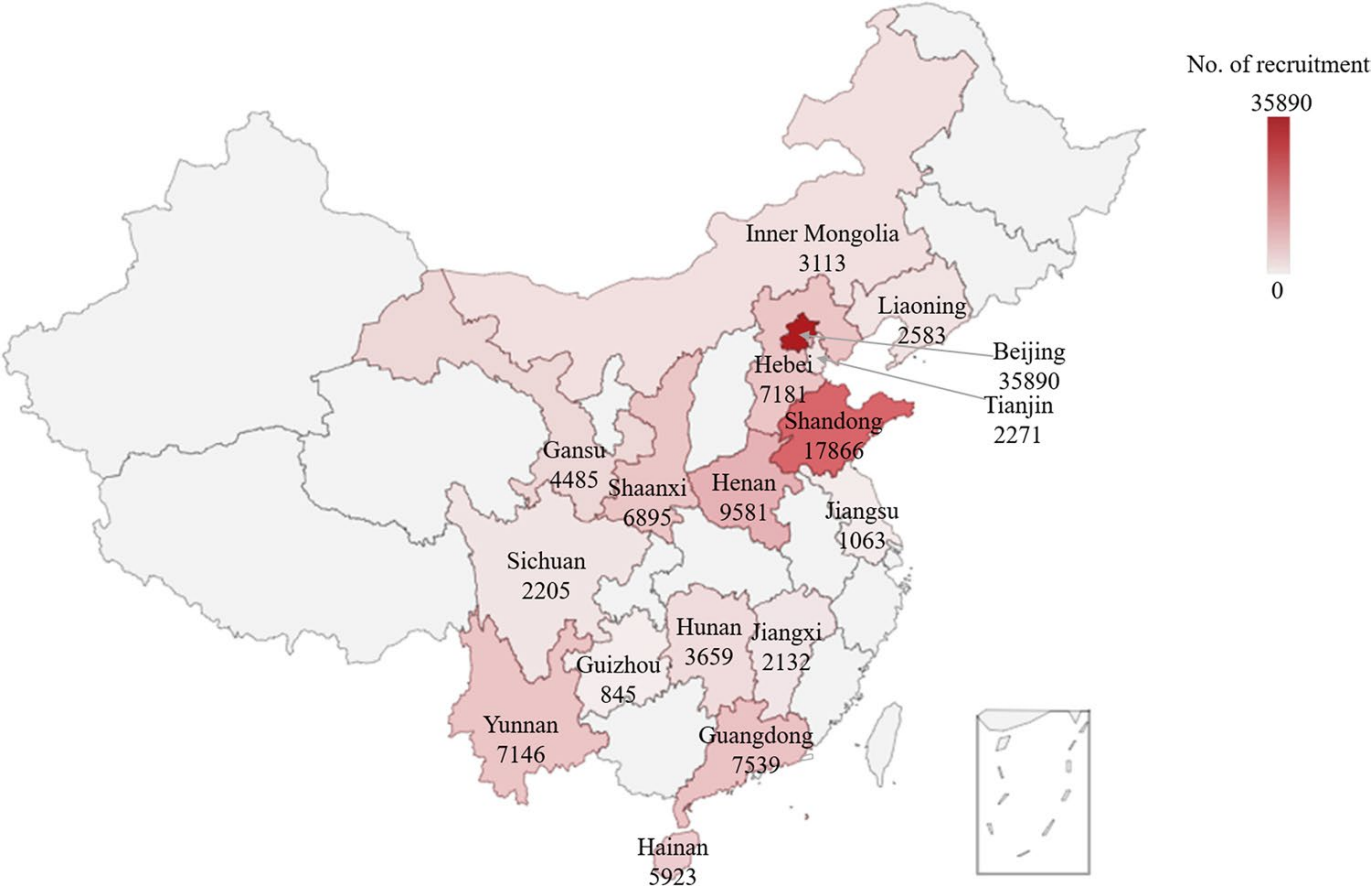
Geographical location of the CBCS eligible study sites. CBCS contains 38 research sites in 17 provinces, cities, autonomous regions and municipalities covering most areas of China

The first two phases have now been completed, with phase 2 finishing, as planned, on 29th February 2020. On that date we had enrolled 120 377 eligible women in early pregnancy (less than 14 weeks of gestation at enrolment), accounting for just over 24.1% of the total target. This recruitment was completed at 38 research sites in 17 provinces, cities, autonomous regions and municipalities covering most areas of China (Fig. 1). Most of these sites are referral hospitals (3A hospitals), with a total of just over 300 000 deliveries annually.

### Followed up strategies

In CBCS, women are enrolled in early pregnancy, at 6–13^+6^ weeks of gestation. At this point, all participants are asked to complete a baseline questionnaire and donate 10 ml of peripheral blood, taken before 13^+6^ weeks of gestation. Clinical laboratory measures are collected for each participant at recruitment (Supplementary table 1). The first and second follow-up visits are undertaken mid-pregnancy at 20–23^+6^ and late pregnancy at 28–33^+6^ weeks of gestation, respectively. For all participants, questionnaires are completed at these two follow-up visits by in-person interviews at their routine prenatal examination. Corresponding clinical laboratory measures are collected at both of these follow-up visits (Supplementary table 1). The third follow-up visit is undertaken after delivery. The clinical information is recorded for all the participants by trained researchers, doctors or nurses.

**Fig. 2:**
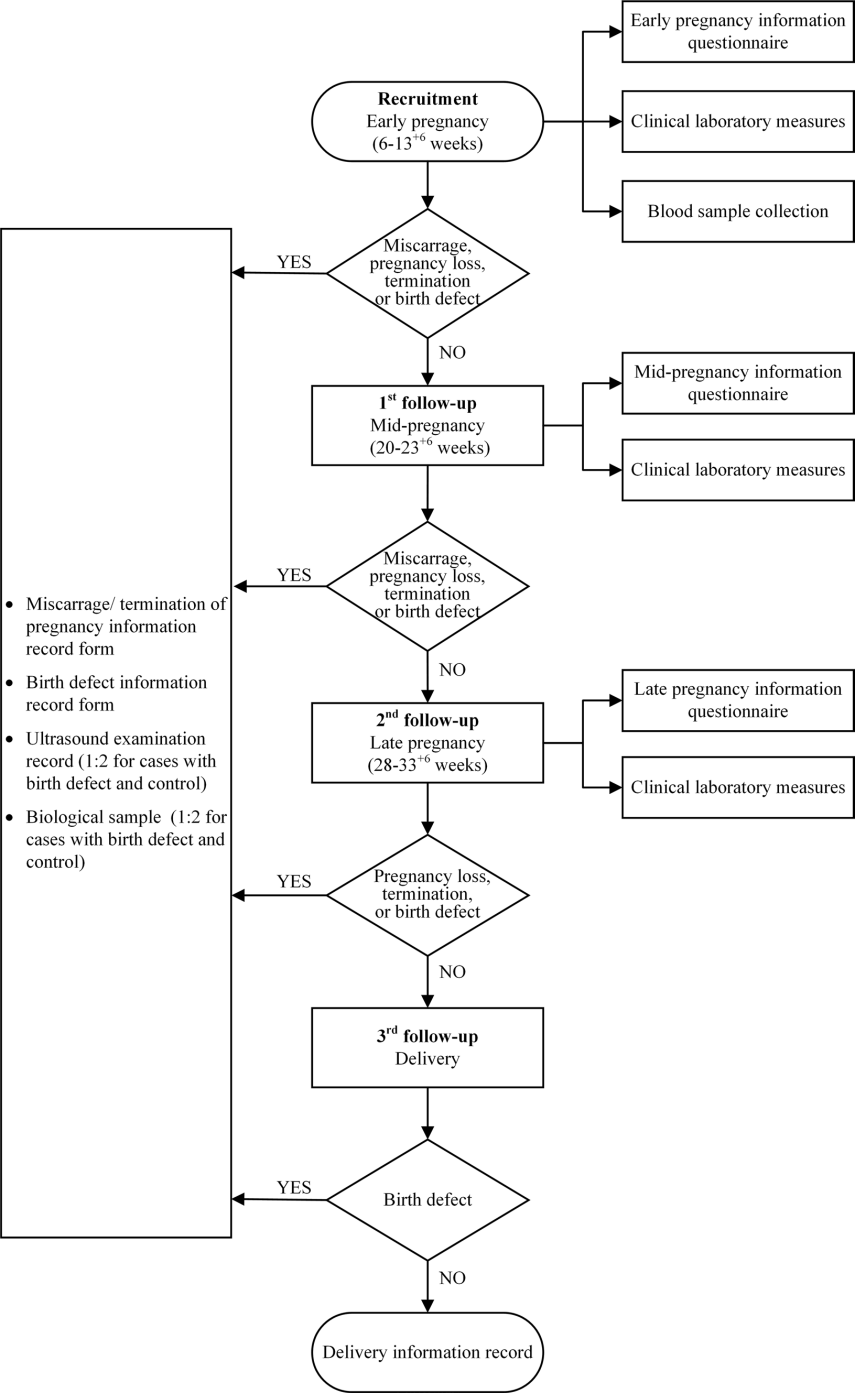
Flow chart of cohort participant recruitment and follow-up

If the participant has a miscarriage in early pregnancy, or a pregnancy loss during mid- or late pregnancy, all clinical information will be recorded by trained researchers, doctors or nurses. If a birth defect is found at any of these stages, clinical information, including ultrasound scan information on fetal defects, is documented, and biological samples collected by specially trained researchers, doctors or nurses according to standard operation procedures and protocols. For each case of a birth defect, two controls are identified with maternal age ± 2 years and gestational age ± 2 weeks. Figure 2 shows the time points of the recruitment and follow-up in CBCS.

### Data collection

The CBCS covers a wide range of areas, including demographic characteristics, health status, reproductive status, prenatal screening, genetic factors, medication exposure and medication, pre-existing chronic disease, nutrition, lifestyle, environment factors and clinical laboratory measurements. All the data collected via the questionnaires, clinical laboratory measurements, standardised ultrasound scans at the enrolment and at follow-up are shown in Table 1. The questionnaire (Questionnaire (Q) 1: 97 questions), completed at enrolment, includes the data on demographic characteristics, outcomes of previous pregnancies, medical history before and during pregnancy, medication, family history on birth defects, occupation, exposures at home and in workplace and lifestyle habits. A food frequency questionnaire (Q2: 70 questions) is required for a representative sample of 80 000 participants. The questionnaires (Q3: 24 questions and Q4: 17 questions) used for the first two follow-up stages, cover the pregnant woman’s health status as well as any prenatal diagnoses. For Q5 (14 questions) data on mode of delivery, perinatal outcomes and birth characteristics (standardised measurement of birth weight, birth length, head circumference, new-born sex, Apgar score and the information of placenta and umbilical cord) are collected at the third follow-up, according to international standards [9]. The information about fathers was embedded in Q1 completed at enrolment, which was filled in by pregnant women, including demographic characteristics, family history of birth defects, smoking status and alcohol use.

**Table 1:** Information collected by questionnaires, physical measurements, laboratory measurements and medical record abstraction, and biological samples collected in the China Birth Cohort study

Stages	Measurements	Methods	Biobank
Early Pregnancy (6–13^+6^ gestation weeks) Enrollment	*Demographic characteristics:* data of birth, ethnic group, education, occupation/employment, income, birthplace; *Health:* height, weight (at preconception and early pregnancy), last menstrual period, blood pressure, health status, age of menarche, menstruation status, medical history, family history of birth defects; *Reproductive status:* method of conception, number of fetuses, reproductive history, history of pregnancy complications; *Lifestyle:* smoking status, alcohol, medication and supplements (folic acid, multi-vitamins), pets exposure; *Environmental exposure: *housing characteristics, decoration, method of heating, use of air purifier, chemical exposure, use of pesticides, occupational exposure, working category, working hours; *Clinical factors and laboratory measurements;* *Diets:* eating habits of the past week, main meals in the past month including staple food, beans, vegetables, fruits, milk, meat, fish products, eggs, snacks, drinking water and soft drinks, cooking oil, dietary supplement;	Structured questionnaire	Whole blood, serum, Chorionic tissue from the fetus*
Mid-pregnancy (20–23^+6^ gestation weeks) Follow-up	Blood pressure, threatened abortion, pregnancy complications (GDM, PIH and Thyroid function); *Prenatal screening:* Down's syndrome screening results, noninvasive prenatal testing, genetic screening for deafness, amniocentesis and other interventional prenatal diagnosis; *Clinical factors and laboratory measurements;*	Structured questionnaire/Physician’s medical records	Amniocyte, cutaneous or muscular tissue from the fetus, placenta, cord blood or umbilical cord tissue*
Late Pregnancy (28–33^+6^ gestation weeks) Follow-up	Blood pressure, threatened premature labor, pregnancy complications (GDM, PIH and Thyroid function); Prenatal screening: amniocentesis and other interventional prenatal diagnosis; *Clinical factors and laboratory measurements;*	Structured questionnaire/Physician’s medical records	Amniocyte, cutaneous or muscular tissue from the fetus, placenta, cord blood or umbilical cord tissue*
Delivery Follow-up	Delivery data, delivery mode, neonatal sex, birth weight, birth length, head circumference, Apgar score, placenta size and shape, umbilical cord length;	Structured questionnaire/Physician’s medical records	Cutaneous or muscular tissue from the fetus, placenta, cord blood or umbilical cord tissue*
Father information	*Demographic characteristics:* data of birth, ethnic group, education, occupation/employment, income; *Health***:** height, weight, family history of birth defects; *Lifestyle:* smoking status, alcohol;	Structured questionnaire	

*Only for cases with birth defects and controls (1:2). PIH: pregnancy induced hypertension. DM: diabetes mellitus

Peripheral blood samples are collected from all participants at enrollment, then processed and stored as whole blood and serum. Chorionic tissue from the fetus are collected from women who have a miscarriage in early pregnancy. Amniocytes are collected for those women opting to have amniocentesis during mid- and late pregnancy. Cutaneous or muscular tissue from the fetus, placenta, cord blood or umbilical cord tissue are collected from women with an induced labour or a birth defect, and cord blood or umbilical cord from controls

All clinical laboratory measures include routine blood tests, as well as blood biochemistry, thyroid function, coagulation function, vitamin level tests, screening for Toxoplasma, Rubella virus, Cytomegalovirus and Herpes virus, maternal serum alpha fetoprotein, oral glucose tolerance test (OGTT) and HbA1c (Supplementary Table 1). Biological samples collected at different periods during pregnancy include peripheral blood samples donated by all participants, and may include amniocytes for those women opting to have amniocentesis, tissue, placenta, cord blood or umbilical cord tissue donated by participants with pregnancy loss or birth defects, and, where appropriate, controls.

### Data management

All baseline, follow-up and outcome data, as well as clinical laboratory measurements and bio-banked samples, are managed through a secure cloud-based platform, including an electronic data capturing (EDC) and a bio-bank system. This platform can combine the information from the two systems automatically and is used to establish, manage and maintain the database. Data can be collected in different ways including on mobile phone, PC or data import. Logic check functions are programmed during data input or capturing in order to avoid data entry errors and missing items, and to ensure integrity and accuracy of the data. The logic validation is not only in each questionnaire, but also operates across different questionnaires for the same woman. Accounts have different levels of permissions for research staff, database administrators and investigators. The platform issues follow-up reminders to both administrators and participants at each follow-up stage. Overall, this cloud-based platform has been designed based on the main principles of data security; ongoing quality control; the ability to share data among different research sites; ease of usability; and extensibility.

The data, including all the measurements in the questionnaire and biological information from each participating centre, are transferred into the central CBCS Database in real time when the information is submitted. Additionally, all collected data is backed up and sent to the central server once a week. The data is managed by professional data administrators and will not be exported until the CBCS steering committee agrees.

Currently, as our study is still in progress, our data cannot be shared externally. However, the data is shared within the team and among participating centres. We will consider the time of data sharing externally when recruitment and follow-up is completed. There will be an application process for use of the data through the completion of a request form which will include the proposed analysis strategy. Applications will be evaluated and once approved by the CBCS committee, data will be shared; results from such analysis must be returned to the database, to allow further sub-analysis.

### Baseline characteristics

The current data are from the pre-planned interim analysis, prior to the third phase of the study, undertaken to ensure proper functioning of all processes, examine follow-up rates, establish whether recruitment of more sites is needed and highlight any problems. As the study is still ongoing, early findings are described below. Table 2 presents the current baseline characteristics of study population. A total of 120 377 pregnancy women have been enrolled, nearly half nulliparous. More than 95% of pregnancies were conceived naturally, while 4.2% used some form of assisted conception. The mean maternal age was 30.08 ± 4.27 and for paternal age it was 31.45 ± 4.93. More fathers were ≥ 36 years old (18.52%) than mothers (11.25%). The age at menarche for most women was 12–14 years, similar to the majority participants in USA, Germany and African-Americans [10–12]. Very few women smoked or consumed alcohol regularly, and more men did so. The prevalence of chronic hypertension and diabetes mellitus before pregnancy was about 0.32% and 0.26% respectively. About 15.44% of women’s pre-pregnancy body mass index (BMI) was over 25 kg/m^2^. However, more men (41.32%) had a BMI above 25 kg/m^2^. The vast majority of participants and fathers were of Han ethnicity (> 93%), which is slightly higher than reported in the *China Statistical Yearbook 2019* (91.5%) [13]. Roughly 65% of participants and fathers had an undergraduate or higher education, and most of the participants were employed (> 75%). Approximately 60% of annual family income was 50 000–200 000 yuan (USD7000-28 000). A small proportion (1.69% and 1.36% of mothers and fathers, respectively) reported a history of any birth defect in their biological families. The prevalence of birth defect in this study was 2.5% and the three leading birth defects were congenital heart defects, urinary system and genital organ malformations, and chromosomal abnormalities.

**Table 2:** Baseline characteristics of participants in the China Birth Cohort study

Characteristics	No. of samples (Mean, 95%CI/median)	Percentage (Standard deviation/IQR)
*Maternal age at pregnancy, years*		
Mean age, 95%CI	30.08, 30.06–30.11	(4.27)
Median age	30.00	(27.00–33.00)
≤ 25	15354	12.75
26–30	53 717	44.62
31–35	37761	31.37
≥ 36	13545	11.25
*Paternal age, years*		
Mean age (SD)	31.45, 31.42–31.48	(4.93)
Median age	31.00	(28.00–34.00)
≤ 25	9888	8.21
26–30	46 744	38.83
31–35	41455	34.44
≥ 36	22290	18.52
*Maternal ethnicity*		
Han	112712	93.63
Others	7665	6.37
*Paternal ethnicity*		
Han	113988	94.69
Others	6389	5.31
*Maternal education*		
< College	27923	23.20
Undergraduate/College	79942	66.41
> Postgraduate or higher	12512	10.39
*Paternal education*		
< College	30696	25.50
Undergraduate/College	77184	64.12
> Postgraduate or higher	12497	10.38
*Maternal occupation*		
Unemployed	29293	24.33
Office worker	9820	8.16
Doctor or medical service personnel	4713	3.92
Banking or commerce or service staff	5970	4.96
Company staff	44145	36.67
Manufactory worker or farmer	1652	1.37
Teacher or researcher or technician	11360	9.44
Military or police	281	0.23
Others	13143	10.92
Maternal annually income, Yuan*		
< 50 000	41195	34.22
50 000–100 000	47277	39.27
100 000–200 000	23796	19.77
200 000–400 000	6328	5.26
400 000–600 000	1192	0.99
> 600 000	589	0.49
Family annually income, Yuan*		
< 50 000	15138	12.58
50 000–100 000	34289	28.48
100 000–200 000	39346	32.69
200 000–400 000	21067	17.50
400 000–600 000	7040	5.85
> 600 000	3497	2.91
Maternal smoking	225	0.19
Paternal smoking	40665	33.78
Maternal alcohol drinking	3950	3.28
Paternal alcohol drinking	38454	31.94
*PIH*		
Yes	384	0.32
No	119993	99.68
*DM*		
Yes	316	0.26
No	120061	99.74
*Maternal biological families with birth defects*	2038	1.69
*Paternal biological families with birth defects* *Age of menarche, years*	1633	1.36
Mean age	13.20, 13.19–13.21	(1.27)
Median age	13.00	(12.00–14.00)
< 12 years	3721	3.09
12–14 years	99492	82.65
> 14 years	17164	14.26
*Pre-pregnancy BMI, kg/m*^*2*^		
Mean BMI	22.06, 22.04–22.08	(4.23)
Median BMI	21.26	(19.53–23.59)
< 18.5	16132	13.40
18.5–25	85662	71.16
25–30	14120	11.73
≥ 30	4463	3.71
*Paternal BMI, kg/m*^*2*^		
Mean BMI	24.96, 24.93–24.99	(4.95)
Median BMI	24.24	(22.13–26.73)
< 18.5	3212	2.67
18.5–25	67430	56.02
25–30	39628	32.92
≥ 30	10107	8.40
*Fertilization way*		
Natural pregnancy	114679	95.27
Artificial insemination	637	0.53
IVF	5061	4.20
*Nulliparous*	58818	48.86

*1 dollar = 7 yuan. PIH: pregnancy induced hypertension. DM: diabetes mellitus. IQR: interquartile range

### Main strengths and weaknesses

The CBCS has a number of strengths. Most important is the very large sample size, the detailed, comprehensive information, and multiple biological samples collected. To our knowledge, this will be the largest birth cohort in the world and will provide adequate power to investigate the causal effects of different exposures on birth defects [14]. The data collected in this study cover environment factors, genetic factors, medication exposure and medication, chronic disease, nutrition and lifestyle. Measuring this multitude of exposures in a single study, coupled with detailed follow up and outcome ascertainment mean that associations can be explored in detail. For example, the relationship between environment (atmosphere, greenness, light, noise, etc.) and teratogenesis, or the associations of the use of drugs with unclear teratogenic effect and birth defects. Moreover, extensive maternal blood samples are collected. Remarkable advances in -omics technologies, including genomics, metabolomics, proteomics and assessment of the microbiome have provided new opportunities for systematic epidemiologic research and further exploration of the mechanism of birth defects. Therefore, integrating-omics and digital technologies, and incorporating a multidisciplinary approach across the life cycle, should be most effective for understanding the factors associated with, and ultimately the prevention, of birth defects.

One weakness of this cohort is the selection bias which had been recognized. However, the main aim of this study is to explore the associations between maternal exposure and birth defects and other adverse outcomes, and the selection is balanced for maternal exposure and the outcomes since the pregnancy outcome is not known at recruitment. Another weakness of this cohort is the self-administered questionnaire at recruitment, with some data based on participant self-evaluation, including health-related variables. However, the questions are presented in simple language and have been piloted; comparison undertaken with forms completed by healthcare staff have demonstrated high concordance. Furthermore, we have collected many different types of biological samples, but we have not started to analyse these. However, genomic sequencing is planned, and we are keen to incorporate emerging new technologies.

In our study, the neonatal outcome at delivery is the final end-point, and no further follow-up of participating mothers and their children is being carried out; this is a weakness when compared to other long-standing birth cohort studies, such as the Danish National Birth Cohort (DNBC) and the Norwegian Mother and Child Cohort Study (MoBa) [14–16]. This is due to financial constraints and a study time-line that funders have implemented; it means that longer-term conditions in the infant, that are not evident at birth, may be under-ascertained. Detailed newborn assessment is being undertaken to mitigate this risk. At the same time we plan to expand the scope of disease and health assessment in future to explore the causal relationship between early life exposures and later health status. In addition, we are aiming to extend our work in order to collect additional information about the father, establish a biological sample database, and develop a long term follow-up plan covering the entire life cycle.

The increasing number of birth cohort studies over recent decades has given opportunities for projects to integrate multiple cohorts, such as the Environmental influences on Child Health Outcomes (ECHO) program which contains 84 observational cohorts and the EU Child Cohort Network which contains 19 pregnancy and childhood cohorts [17, 18]. Combining data will make it possible to identify smaller effect estimates, and search for differences in risk factors across countries. This will enable better research into causal understanding and modelling of life course health trajectories. To maximize the benefits of our research, we are already forming links to collaborate with other birth cohort studies, and our data will ultimately be made accessible to other researchers.

## Supplementary Information

Below is the link to the electronic supplementary material.

Supplementary file1 (DOCX 16 KB)
