# Supplementary material for: The China birth cohort study (CBCS)
Source: Eur J Epidemiol. 2022 Feb 11;37(3):295–304. doi: 10.1007/s10654-021-00831-8 (PMC9110496; doi:10.1007/s10654-021-00831-8)
Supplement: Supplementary file 1 — Supplementary file1 (DOCX 16 KB) [file 10654_2021_831_MOESM1_ESM.docx]

**Supplementary table 1**

Laboratory measures in blood tests in CBCS.

| Variables | 6-13^+6^ weeks gestation weeks  (Recruitment) | 20-23^+6^ weeks gestation weeks  (Follow-up) | 28-33^+6^ weeks gestation weeks  (Follow-up) |
| --- | --- | --- | --- |
| Routine blood test | $\surd$ | $\surd$ | $\surd$ |
| Blood biochemical test | $\surd$ | $\surd$ | $\surd$ |
| Thyroid function test | $\surd$ | $\surd$ | $\surd$ |
| Coagulation function test | $\surd$ |  | $\surd$ |
| Vitamin test | $\surd$ |  |  |
| Toxoplasma | $\surd$ |  |  |
| Rubellavirus | $\surd$ |  |  |
| Cytomegalovirus | $\surd$ |  |  |
| Herpesvirus | $\surd$ |  |  |
| Alpha fetoprotein detection |  | $\surd$ |  |
| Oral glucose tolerance test (OGTT) |  |  | $\surd$ |
| HgbA1c* |  |  | $\surd$ |

*If OGTT is abnormal, HgbA1c is required.

Routine blood test includes white blood cell, red blood cell, haemoglobin, platelet counts, hematocrit, granulocyte and lymphocyte.

Blood biochemical test includes fasting blood glucose, triglycerides, total cholesterol, high-density lipoprotein cholesterol, low-density lipoprotein cholesterol, uric acid, blood urea nitrogen, alanine aminotransferase, aspartate amino transferase and albumin.

Thyroid function test includes total thyroxine, total triiodothyronine, thyroxine binding globulin, free triiodothyronine, free thyroxine, thyroid-stimulating hormone and thyroid peroxidase antibody.

Coagulation function test includes thrombin time, prothrombin time, fibrinogen, D-dimer and partial thromboplastin time.

Vitamin test includes vitamin A, vitamin D and vitamin E.
